# Causal effects of endometriosis on serum 25-hydroxyvitamin D: Evidence from Mendelian randomization study

**DOI:** 10.1097/MD.0000000000048562

**Published:** 2026-05-08

**Authors:** Hongkun Jiang, Yinghua Tang, Ximei Huang, Zila Wang, Faquan Lin

**Affiliations:** a Department of Clinical Laboratory, The First Affiliated Hospital of Guangxi Medical University, Key Laboratory of Clinical Laboratory Medicine of Guangxi Department of Education, Nanning, Guangxi, China; b Department of Clinical Laboratory, Liuzhou People’s Hospital, Liuzhou, Guangxi, China.

**Keywords:** endometriosis, instrumental variant, Mendelian randomization, serum 25-hydroxyvitamin D, single-nucleotide polymorphism

## Abstract

The potential bidirectional causal relationship between endometriosis and serum 25-hydroxyvitamin D has been previously investigated. Nevertheless, the results could be impacted by confounding factors and reverse causality because of the nature of observational research. Mendelian randomization (MR) circumvents the drawbacks of observational studies by providing estimates of unconfounded causal effects. We used bi-directional MR analysis to determine whether serum 25-hydroxyvitamin D had a causal influence on endometriosis and vice versa. Additionally, using heterogeneity and sensitivity analyses, we explored the heterogeneity and pleiotropy of effect estimates. Similarly, we investigated the causal correlation between endometriosis and 25-hydroxyvitamin D through reverse-direction MR. In forward-direction MR, we selected 95 single-nucleotide polymorphisms (SNPs) as instrumental variants (IVs) to examine the potential causal influence between 25-hydroxyvitamin D and endometriosis. And we selected 9 SNPs as IVs for reverse-direction MR analyses to examine whether endometriosis has a causal influence on serum 25-hydroxyvitamin D. We found suggestive evidence that endometriosis contributes to higher serum 25-hydroxyvitamin D (β: 0.010, 95% CI: 0.002–0.02, *P* = .016). Our data suggest that serum 25-hydroxyvitamin D levels may be a consequence of endometriosis rather than a causal risk factor, and its potential role in the disease process warrants further investigation.

## 1. Introduction

Endometriosis (EMs) is a clinical condition characterized by a chronic inflammatory response to estrogen that mostly affects pelvic tissues, including the ovaries.^[1]^ Endometriosis is a common disorder resulting infertility in women.^[2]^ Worldwide, 5% to 10% of women who are fertile are affected by endometriosis.^[3]^ Additionally, the currently available pharmacological and surgical therapies are ineffective.^[4,5]^ Vitamin D is a fat-soluble vitamin, with 25-hydroxyvitamin D as its primary circulating form.^[6]^ It may play roles in reproductive health, including recurrent pregnancy loss and dysmenorrhea.^[7-9]^

A prospective cohort study suggested that higher serum 25-hydroxyvitamin D levels and increased consumption of dairy products are connected with a lower risk of endometriosis.^[10,11]^ Consuming ice cream and yogurt during youth may lower the likelihood of emergence and growth of endometriosis later in life.^[12]^ In contrast, a case-control study suggested that consumption of milk was not significantly related to endometriosis.^[13]^ As shown above, the results of research on the correlation between 25-hydroxyvitamin D and endometriosis have been conflicting. Thus, further investigation is warranted.

Furthermore, observational studies cannot infer causality for the function of 25-hydroxyvitamin D in the occurrence and development of endometriosis. Potential drawbacks of these studies include reverse causality and confounding factors, which might mask the underlying causal relationship. The discovery of genetic variants that determine serum 25-hydroxyvitamin D levels may assist in obtaining more accurate estimates of the relationship with minimized bias due to confounding.

Genome-wide association studies (GWAS) are a powerful tool for identify millions of SNPs that mediate susceptibility to certain disorders in the past 10 years. Cohort or case-control research designs are used in GWAS to evaluate complex features, and the findings are typically posted in open-access databases, such as the Integrative Epidemiology Unit GWAS database or the GWAS catalog.

Mendelian randomization (MR) is a new statistical strategy that using single-nucleotide polymorphisms (SNPs) as instrumental variables (IVs) for the identification of potential causative influence between exposure and outcome.^[14-16]^ This approach can be used to examine these publicly available GWAS results.^[17]^ Therefore, in this study, we used MR to explore the causative influence between 25-hydroxyvitamin D and endometriosis.

## 2. Materials and methods

### 2.1. Ethical approval

This study analyzed publicly available, de-identified summary-level data from genome-wide association studies (GWAS). Therefore, separate ethical approval was not required for this specific analysis. The original GWAS studies obtained ethical approval from their respective institutional review boards (IRBs) and informed consent from all participants.

### 2.2. Mendelian randomization

MR is a valuable tool for determining causation in observational data.^[18]^ Compared with traditional observational study design, MR can avoid the problems of confounding effects or reverse causality. It permits the testing of causation, thereby potentially helping to understand and prevent the adverse consequences of modifiable exposures on human health.^[17,19,20]^

With ever-increasing in sample sizes of GWAS, genetic variants linked to 25-hydroxyvitamin D and endometriosis have been recently recognized.^[21]^ We are unaware of any studies that have attempted to use MR to explore the potential causative influence between 25-hydroxyvitamin D and endometriosis. In this research, we performed a MR study (first MR analysis) to determine genetically proxied serum 25-hydroxyvitamin D causally affected the risk of endometriosis. Furthermore, in the second MR analysis, we examined whether endometriosis exerts the same causal effect on serum 25-hydroxyvitamin D.

### 2.3. GWAS data source

This bidirectional MR study relied on GWAS. To perform MR analyses, we selected SNPs as instrumental variants (IVs) for serum 25-hydroxyvitamin D (ebi-a-GCST90000618; n = 4,96,946) and endometriosis (finn-b-N14_ENDOMETRIOSIS; n = 8288).^[22]^ Subsequently, we chose SNPs from serum 25-hydroxyvitamin D and endometriosis studies that only enrolled individuals of European ancestry. We preferred data sources with large sample sizes, and solely female individuals. Of note, female-only GWAS have not been conducted thus far.

### 2.4. Genetic variables

We carried out a number of quality control procedures for the selection of eligible IVs. Specifically, after identifying the SNPs from the GWAS, the clump function was used to ensure their independence. To determine the *R*^2^-value, we used the standard formula for a continuous trait: *R*^2^ = 2 × EAF × (1 − EAF) × β^2^. Thereafter, to meet the first MR assumption, we used the expected value of the *F*-statistic to assess the overall efficacy of the 95 chosen SNPs in explaining 25-hydroxyvitamin D.^[23,24]^ Similarly, 9 chosen SNPs were used as IVs for our reverse MR.

Menstrual cycle length, body mass index (BMI) and body weight are endometriosis-relevant features that may be confounders in the correlation between 25-hydroxyvitamin D and endometriosis.^[25]^ In order to meet the second MR assumption, we excluded the IVs that arrogated these endometriosis-relevant features at *R*^2^ > 0.80 using PhenoScannerV2 database.^[26,27]^ We specifically checked for associations with potential confounders such as BMI, smoking, alcohol consumption, and sun exposure. Next, we harmonized the 25-hydroxyvitamin D and endometriosis data by eliminating palindromic SNPs. A palindromic SNP is one whose potential alleles couple with each other within the double helix structure; allele frequencies between 0.01 and 0.30 were referred to as intermediate allele frequencies.

### 2.5. Estimation of causal effect

We created a list of SNPs and performed MR analysis using Inverse-variance weighted (IVW) method. Its technique considers single SNP to be a genuine natural experiment, computes the Wald ratio and then integrates the findings using an IVW meta-analysis methodology. And the estimated slope parameter obtained from this method may indicate the causal influence of the exposure on the result. In addition, we calculated the odds ratios (OR) for effect estimates and 95% confidence intervals (CIs). Hence, IVW estimation may yield the most precise results. We also performed MR analysis using multiple methods to make conclusions more reliable, such as weighted median (WM), MR-Egger, simple mode, and weighted mode methods.

We performed heterogeneity and pleiotropy analyses to assess the robustness of the results using the “leave-one-out” sensitivity analysis and MR-PRESSO analysis.^[18,28,29]^ *Q*-statistics and *I*^2^-values analyze can be valuable in assessing the pleiotropy of IVs.^[30]^

### 2.6. Statistical analysis

**Figure 1.**
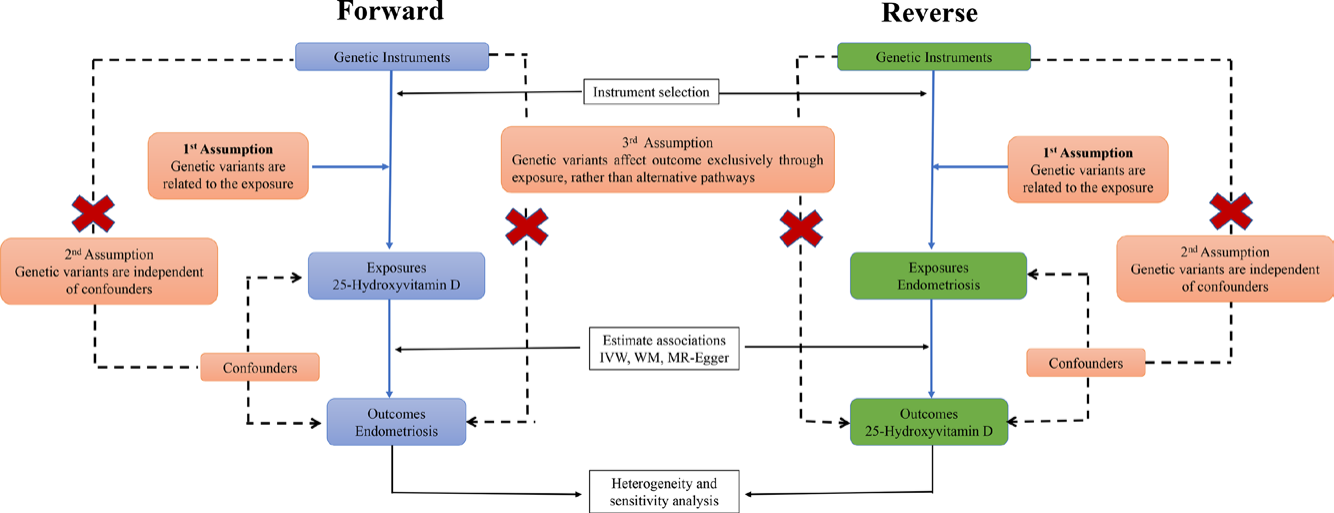
Flowchart of the 2SMR analysis. Genetic variants were used as instrumental variables (IVs) to assess the causal effect of an exposure (forward direction: 25-hydroxyvitamin D; reverse direction: endometriosis 25-hydroxyvitamin) on an outcome (forward direction: endometriosis; reverse direction: 25-hydroxyvitamin D). 2SMR, 2-sample Mendelian randomization, IV = instrumental variable, IVW = inverse-variance weighted, MR = Mendelian randomization, WM = weighted median.

We obtained MR estimates using the “devtools,” “MRCIEU/TwoSampleMR,” “MR-PRESSO,” and “LDlinkR” functions in R statistical software (version 4.2.2). Of note, *P*-values < .05 denoted a causative relationship between exposures and outcomes. Figure 1 displays the 2-sample Mendelian randomization (2SMR) analysis flowchart.

## 3. Results

### 3.1. Effect of serum 25-hydroxyvitamin D on endometriosis

In our first MR, including 95 SNPs as IVs, we estimated the causative influence between 25-hydroxyvitamin D and endometriosis. Information on the 95 SNPs with 25-hydroxyvitamin D and endometriosis are presented in Table S1, Supplemental Digital Content. Cumulative *F*-statistic value for these 95 selected SNPs was 36.87, indicating that the entire collection of 95 SNPs satisfied the highly relevant assumption of MR.

**Figure 2.**
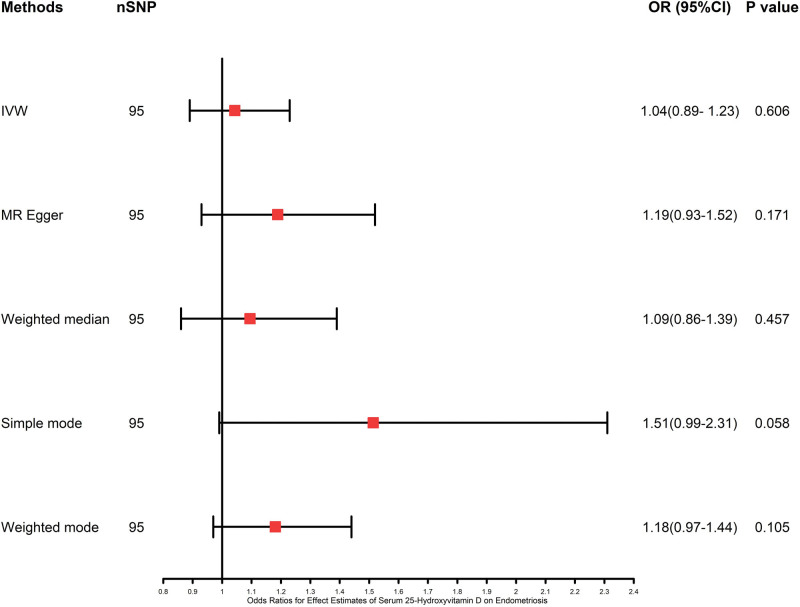
Mendelian randomization analysis of serum 25-hydroxyvitamin D to endometriosis using different methods. CI = confidence interval, IVW = inverse-variance weighted, MR = Mendelian randomization, OR = odds ratio, SNP = single-nucleotide polymorphism.

Table 1 and Figure 2 show the results of the IVW (OR: 1.040, 95% CI: 0.890–1.230, *P* = .606), MR-Egger (OR: 1.190, 95% CI: 0.930–1.520, *P* = .171), weighted median (OR: 1.090, 95% CI: 0.860–1.390, *P* = .457), simple mode (OR: 1.510, 95% CI: 0.990–2.310, *P* = .058), and weighted mode (OR: 1.180, 95% CI: 0.970–1.440, *P* = .105) methods using 95 SNPs. The results of IVW method did not reveal a causative influence of 25-hydroxyvitamin D on endometriosis. Results of MR-Egger, weighted mode, weighted median and simple mode were consistent.

**Table 1:** MR analysis for the causality of serum 25-hydroxyvitamin D with the risk of endometriosis.

Exposure/outcome	Nsnp	Methods	OR (95% CI)	SE	*P*-value	Pleiotropy			Heterogeneity			*R* ^2^	*F*
						MR-Egger regression			Cochran *Q*	*I* ^2^	*P*-value		
						Egger intercept	SE	*P*-value					
Serum 25-hydroxyvitamin D/endometriosis	95	IVW	1.04 (0.89–1.23)	0.08	.606	−0.005	0.003	.171	113.770	0.170	.081	0.007	36.870
		MR Egger	1.19 (0.93–1.52)	0.13	.171								
		Weighted median	1.09 (0.86–1.39)	0.12	.457								
		Simple mode	1.51 (0.99–2.31)	0.21	.058								
		Weighted mode	1.18 (0.97–1.44)	0.10	.105								

CI = confidence interval, *I*^2^ = heterogeneity index, IVW = inverse-variance weighted, MR = Mendelian randomization, Nsnp = number of single-nucleotide polymorphisms, OR = odds ratio; SE = standard error.

**Figure 3.**
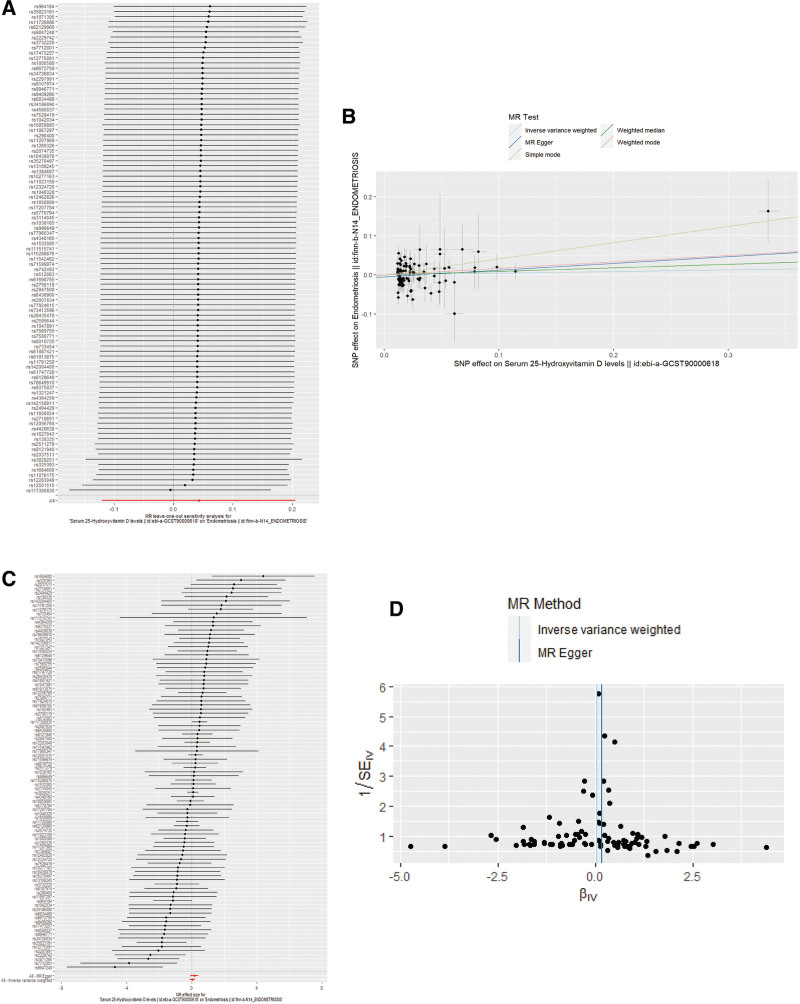
Effect of serum 25-hydroxyvitamin D on endometriosis. (A) Leave-one-out plot. (B) Scatter plot. (C) Forest plot. (D) Funnel plot. MR = Mendelian randomization, SE = standard error, SNP = single-nucleotide polymorphism.

In the test of heterogeneity, *Q*-statistics and *I*^2^-values did not reveal significant heterogeneity (*Q* = 113.77, *I*^2^ = 0.171, *P* = .081). We use “leave-one-out” analysis to identify pleiotropic variants. The results revealed that removal of any SNP from the principal MR analysis would not alter the MR estimates (Fig. 3A). In addition, the MR-Egger analysis did not reveal a pleiotropic effect (intercept = −0.005, SE = 0.003, *P* = .171; Fig. 3B). Interestingly, the outlier-corrected MR-PRESSO technique failed to identify any outlying SNP (causal estimate = 0.043, standard deviation = 0.082, *T*-statistic = 0.519, *P* = .605). Plots of the causal effect estimate and sensitivity are shown in Figure 3. Therefore, our primary MR study was reliable, as demonstrated by the lack of observable heterogeneity and pleiotropy. In conclusion, our primary 2SMR analyses with 5 methods consistently demonstrated no evidence of causative influence between 25-hydroxyvitamin D and endometriosis.

### 3.2. Effect of endometriosis on serum 25-hydroxyvitamin D

In our second MR, 9 SNPs were selected for reverse-direction MR analyses to examine whether endometriosis has a causal influence on 25-hydroxyvitamin D levels. In the PhenoScanner search, no SNP was found to be associated with 25-hydroxyvitamin D. Table S2, Supplemental Digital Content shows the information on SNPs linked to endometriosis and their association with 25-hydroxyvitamin D. The cumulative *F*-statistic value for these 9 selected SNPs was 53.60, indicating that these SNPs were a suitable endometriosis proxy to determine the causative influence.

**Figure 4.**
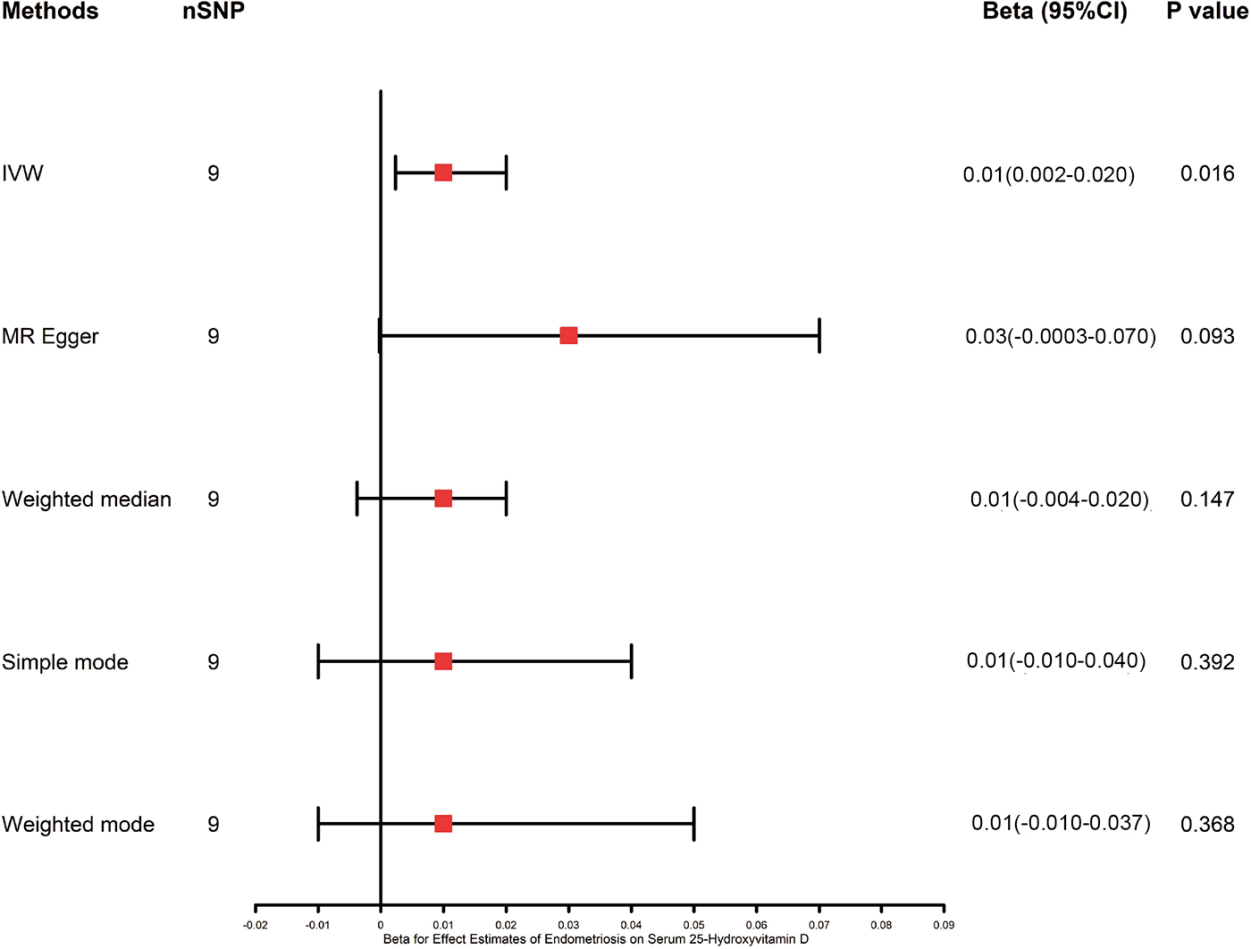
Mendelian randomization analysis of endometriosis to serum 25-hydroxyvitamin D using different methods. CI = confidence interval, IVW = inverse-variance weighted, MR = Mendelian randomization, SNP = single-nucleotide polymorphism.

**Figure 5.**
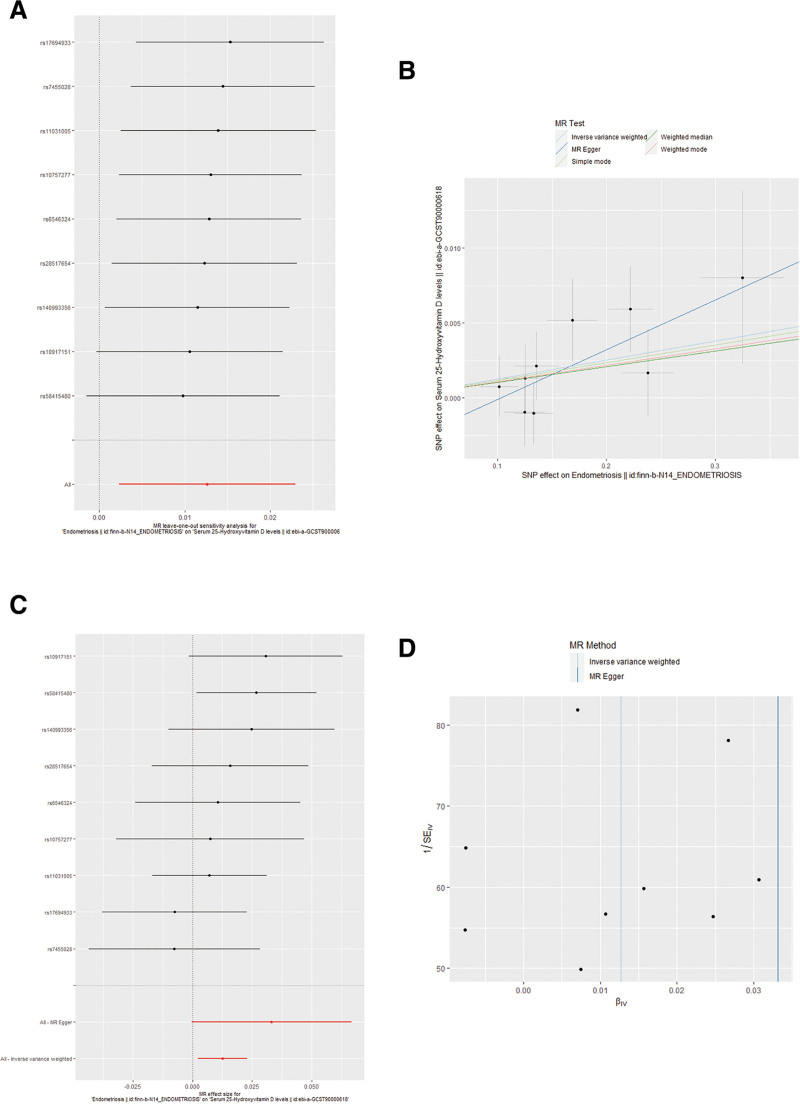
Effect of endometriosis on serum 25-hydroxyvitamin D levels. (A) Leave-one-out plot. (B) Scatter plot. (C) Forest plot. (D) Funnel plot. MR = Mendelian randomization, SE = standard error, SNP = single-nucleotide polymorphism.

While the IVW method suggested a potential causal effect of endometriosis on serum 25-hydroxyvitamin D (β: 0.010, 95% CI: 0.002–0.020, *P* = .016; Table 2), more robust methods such as MR-Egger (β: 0.030 95% CI: −0.0803 to 0.070, *P* = .093), weighted median (β: 0.010, 95% CI: −0.004 to 0.020, *P* = .147), simple mode (β: 0.010, 95% CI: −0.010 to 0.040, *P* = .392), and weighted mode (β: 0.010, 95% CI: −0.010 to 0.040, *P* = .368) did not yield significant results (Table 2 and Fig 4), indicating that this finding should be interpreted with caution. As visually apparent in the scatter plot (Fig. 5B) and method comparison (Fig. 4), the significant effect was driven by the IVW method, while the MR-Egger and Weighted Median estimates were consistent with a null effect, suggesting the finding may not be robust to potential pleiotropy.

**Table 2:** MR analysis for the causality of endometriosis with the levels of serum 25-hydroxyvitamin D.

Exposure/outcome	Nsnp	Methods	β (95% CI)	SE	*P*-value	Pleiotropy			Heterogeneity			*R* ^2^	*F*
						MR-Egger regression			Cochran *Q*	*I* ^2^	*P*-value		
						Egger intercept	SE	*P*-value					
Endometriosis/serum 25-hydroxyvitamin D levels	9	IVW	0.010 (0.002–0.020)	0.004	.016	−0.003	0.003	.246	6.120	0.340	.630	0.001	53.600
		MR Egger	0.030 (−0.0003 to 0.070)	0.020	.093								
		Weighted median	0.010 (−0.004 to 0.020)	0.006	.147								
		Simple mode	0.010 (−0.010 to 0.040)	0.013	.392								
		Weighted mode	0.010 (−0.010 to 0.037)	0.010	.368								

The significant effect is driven by IVW, with methodological discrepancy across more robust methods.

CI = confidence interval, *I*^2^ = heterogeneity index, IVW = inverse-variance weighted, MR = Mendelian randomization, Nsnp = number of single-nucleotide polymorphisms, SE = standard error.

MR-Egger regression did not detect a pleiotropic effect (intercept = −0.003, SE = 0.003, *P* = .246; Fig. 5B). Likewise, no heterogeneity was found using *Q*-statistics and *I*^2^-values (*Q* = 6.120, *I*^2^ = 0.340, *P* = .630). Plot for the “leave-one-out” sensitivity analysis indicated that the causal correlation between genetically predicted endometriosis on serum 25-hydroxyvitamin D was not substantially driven by any single SNP (Fig. 5A). A scatter plot showing the relationship between the SNP for endometriosis and the SNP for 25-hydroxyvitamin D using several MR techniques and sensitivity analysis (Fig. 5B). Forest plot indicate a causal relationship between genetically proxied endometriosis and the 25-hydroxyvitamin D levels based on IVW methods (*P* < .05) using 9 SNPs (Fig. 5C). In conclusion, our reverse 2SMR analyses provide suggestive but not robust evidence of a potential causal influence of endometriosis on serum 25-hydroxyvitamin D levels.

## 4. Discussion

Endometriosis is a common clinical gynecological disorder in women. In this study, we performed systemic 2SMR analysis using a stronger genetic instrument versus conventional methods to identify any links between endometriosis and serum 25-hydroxyvitamin D. Furthermore, our bidirectional MR analysis did not detect a causal influence of 25-hydroxyvitamin D on the development of endometriosis; however, we found suggestive evidence that endometriosis may be associated with slightly elevated 25-hydroxyvitamin D levels.

### 4.1. Causative influence of serum 25-hydroxyvitamin D on endometriosis

Several observational studies have shown the connection between 25-hydroxyvitamin D and the development of endometriosis. According to a prospective cohort research study, higher 25-hydroxyvitamin D levels are associated with a lower risk of endometriosis.^[10]^ In another prospective cohort research suggested that consumption of dairy in adolescence, namely the intake of yogurt and ice cream, might be a method to lower the risk of developing endometriosis.^[12]^ Additionally, a systematic literature review uncovered that dietary consumption of vitamin D and the concentration of serum 25-hydroxyvitamin D may affect the risk of endometriosis.^[31]^ However, these observational studies may be confined by potential limitations (e.g., reverse causality and confounding factors), which obscure the true causal relationship.

In our study, the findings of our forward-direction MR analysis were inconsistent with the outcomes of earlier observational investigations. We did not detect any causative influence of serum 25-hydroxyvitamin D on endometriosis.

Our MR analysis helps clarify the relationship suggested in previous observational studies and found no evidence supporting a causal effect of serum 25-hydroxyvitamin D on endometriosis. The differences between the results obtained from observational and MR investigations can be attributed to the intrinsic advantage of the latter over the former, as well as methodological flaws in observational research.

### 4.2. Causative influence of endometriosis on serum 25-hydroxyvitamin D

Clinical research performed by Somigliana et al demonstrated that endometriosis was linked to higher levels of serum 25-hydroxyvitamin D.^[32]^ Another clinical investigation on endometriosis patients discovered higher levels of vitamin D-binding protein (VDBP) in their urine.^[33]^ However, according to an in vitro research study, women with severe endometriosis had considerably lower blood levels of 25-hydroxyvitamin D than controls and those with moderate endometriosis.^[34]^ Nonetheless, whether endometriosis is responsible for the higher levels of serum 25-hydroxyvitamin D remains largely unexplored.

Recently, a bidirectional MR study by Pan D and colleagues published in Reproductive Sciences investigated the causal relationship between serum 25-hydroxyvitamin D levels and endometriosis. Their analysis suggested a potential bidirectional association between vitamin D status and the risk of endometriosis.^[35]^ While their findings provide important insights into the interplay between vitamin D metabolism and endometriosis, our study differs in several aspects. First, we used a different GWAS dataset for serum 25-hydroxyvitamin D that included a larger sample size, which may improve the statistical power and robustness of the MR analysis. Second, although both studies employed bidirectional MR approaches, our analysis further evaluated the potential causal influence of endometriosis on circulating vitamin D levels using additional sensitivity analyses. Therefore, our results provide complementary evidence supporting the possible interaction between endometriosis and vitamin D metabolism.

Our reverse-direction MR research provides suggestive evidence for a causal effect of endometriosis on serum 25-hydroxyvitamin D. If there is a causal effect, it is likely small and may reflect the inflammatory state of the disease. In line with clinical trial that human cycling endometrium may be one of the extrarenal locations capable of vitamin D synthesis.^[8]^ The potential effect of endometriosis on 25-hydroxyvitamin D may be related to inflammation, as endometriosis is an inflammatory condition where macrophages mediate responses.^[36]^ For instance, activated macrophages may produce 25-hydroxyvitamin D that spills into circulation.^[37]^ A previous MR study indicated high levels of macrophage colony-stimulating factor (MCSF) are associated with increased risks of endometriosis.^[38]^ However, this mechanism is speculative and should be framed as a hypothesis.

Our MR analyses were based on the PhenoScannerV2 database; hence, they are superior to existing observational research in terms of significantly reduced confounding bias.

### 4.3. Heterogeneity and pleiotropy

In addition, several heterogeneity and pleiotropy analyses were conducted to investigate potential pleiotropy. These analyses showed no significant heterogeneity or pleiotropy in the forward MR estimates (e.g., *Q* = 113.77, *I*^2^ = 0.171, *P* = .081; MR-Egger intercept = −0.0051, *P* = .171). For the reverse MR, no heterogeneity was detected (*Q* = 6.120, *I*^2^ = 0.340, *P* = .630), and MR-Egger regression indicated no directional pleiotropy (intercept = −0.003, *P* = .246).

The reverse MR finding showed conflicting results between methods, with only the IVW method being significant while more robust methods (MR-Egger and weighted median) were not. This implies that the evidence for a causal effect is suggestive but not conclusive, potentially due to pleiotropy or other biases, and warrants cautious interpretation and replication in future studies.

### 4.4. Implications

Our bidirectional MR analysis demonstrated no causal influence of 25-hydroxyvitamin D on endometriosis, with suggestive evidence for the reverse. These findings suggest that serum 25-hydroxyvitamin D levels may be a consequence of endometriosis rather than a causal risk factor. The present results may be important for public health because they could be used as a basis for further research on endometriosis and serum 25-hydroxyvitamin D. In addition, they provide new information regarding the presumed differences in the etiologies of endometriosis and alterations in serum 25-hydroxyvitamin D.

### 4.5. Strength and limitations

To our knowledge, this study provides additional MR evidence regarding the relationship between serum 25-hydroxyvitamin D and endometriosis using updated GWAS datasets. In comparison to observational studies, the enormous sample size of GWAS summary datasets used in MR considerably reduced the degree of reverse causation and confounding bias.

However, our research is also characterized by several limitations. First, we did not provide a physiological explanation regarding the correlation between 25-hydroxyvitamin D and endometriosis. Second, our investigation focused on individuals of European ancestry; consequently, the results may not accurately reflect the relationship between 25-hydroxyvitamin D and endometriosis in individuals of different ancestries, such as Asians and Blacks. Thus, further research is warranted to generalize the present findings to non-European populations. Third, although endometriosis exclusively affects women, the serum 25-hydroxyvitamin D GWAS utilized in this investigation included both male and female participants. Therefore, if new GWAS on serum 25-hydroxyvitamin D in females become available, it would be intriguing to conduct a similar study. Fourth, the relatively small sample size of the endometriosis GWAS (n = 8288) may lead to weak instrument bias, although *F*-statistics were strong (>10). Finally, the credibility of findings may also be somewhat compromised by any error in measurement. Thus, additional research on this topic should be conducted when larger and more comprehensive datasets become available to the general public.^[39,40]^

Therefore, the causal estimate for this direction should be considered preliminary and requires validation in future studies with stronger genetic instruments and consistent results across MR methods.

## 5. Conclusion

Our bidirectional MR analysis produced evidence indicating that 25-hydroxyvitamin D is not causally associated with the risk of developing endometriosis. However, there is suggestive evidence that endometriosis may lead to slightly elevated serum 25-hydroxyvitamin D levels, though this finding is not robust across methods and requires further investigation.

## Acknowledgments

The authors thank all the participants and researchers of the original genome-wide association studies who contributed and shared the summary statistics. We specifically acknowledge the MRC-IEU UK Biobank GWAS pipeline for the 25-hydroxyvitamin D data (accession: ebi-a-GCST90000618) and the FinnGen consortium for the endometriosis data (release R9, finn-b-N14_ENDOMETRIOSIS).

## Author contributions

**Conceptualization:** Hongkun Jiang, Faquan Lin.

**Data curation:** Ximei Huang, Zila Wang.

**Formal analysis:** Hongkun Jiang.

**Software:** Hongkun Jiang, Yinghua Tang, Faquan Lin.

**Visualization:** Hongkun Jiang, Yinghua Tang, Faquan Lin.

**Writing – original draft:** Hongkun Jiang.

**Writing – review & editing:** Hongkun Jiang, Faquan Lin.




