# Supplementary material for: Causal effects of endometriosis on serum 25-hydroxyvitamin D: Evidence from Mendelian randomization study
Source: Medicine (Baltimore). 2026 May 8;105(19):e48562. doi: 10.1097/MD.0000000000048562 (PMC13166774; doi:10.1097/MD.0000000000048562)
Supplement: Supplementary file 1 [file medi-105-e48562-s001.docx]

**TABLE S1. Single-Nucleotide Polymorphisms (SNPs) for 25-Hydroxyvitamin D Levels and Their Association with Endometriosis**

| **Target SNP** | **Chr** | **Effect Allele (Alternative)** | **Association with** **25-Hydroxyvitamin D** **levels** | | | | **MAF** | **R^2^** | **Association with** **Endometriosis** | | | |
| --- | --- | --- | --- | --- | --- | --- | --- | --- | --- | --- | --- | --- |
|  |  |  | **β** | **SE** | **EAF** | ***p*** |  |  | **β** | **SE** | **EAF** | ***p*** |
| rs10277163 | 7 | G(A) | -0.0143 | 0.0024 | 0.2547 | 1.08×10^-9^ | 0.2576 | 0.00002862 | 0.0121 | 0.0210 | 0.2259 | 0.5653 |
| rs1038165 | 12 | T(C) | 0.0115 | 0.0021 | 0.5795 | 2.15×10^-8^ | 0.4444 | 0.00003116 | 0.0016 | 0.0177 | 0.5734 | 0.9286 |
| rs1042034 | 2 | T(C) | -0.0151 | 0.0025 | 0.7921 | 1.45×10^-9^ | 0.2121 | 0.00002462 | 0.0198 | 0.0198 | 0.7315 | 0.3165 |
| rs10438978 | 18 | C(T) | -0.0172 | 0.0026 | 0.8203 | 7.34×10^-11^ | 0.1667 | 0.00002372 | 0.0148 | 0.0230 | 0.8221 | 0.5192 |
| rs1047891 | 2 | A(C) | -0.0134 | 0.0022 | 0.3171 | 7.96×10^-10^ | 0.2879 | 0.00003116 | -0.0097 | 0.0188 | 0.3232 | 0.6070 |
| rs1048328 | 19 | A(G) | 0.0313 | 0.0037 | 0.0798 | 5.58×10^-17^ | 0.0808 | 0.00002096 | -0.0082 | 0.0293 | 0.1009 | 0.7788 |
| rs10859995 | 12 | C(T) | -0.0436 | 0.0021 | 0.5798 | 4.60×10^-100^ | 0.4293 | 0.00044460 | 0.0032 | 0.0185 | 0.6622 | 0.8628 |
| rs11023159 | 11 | C(T) | 0.0482 | 0.0057 | 0.0325 | 3.73×10^-17^ | 0.0303 | 0.00000839 | -0.0198 | 0.0512 | 0.0309 | 0.6992 |
| rs11076175 | 16 | G(A) | 0.0229 | 0.0027 | 0.1757 | 9.64×10^-18^ | 0.1919 | 0.00004592 | 0.0409 | 0.0233 | 0.1711 | 0.0789 |
| rs111515741 | 11 | A(G) | -0.0487 | 0.0078 | 0.0173 | 3.95×10^-10^ | 0.0239 | 0.00000367 | -0.0654 | 0.1428 | 0.0039 | 0.6470 |
| rs11207969 | 1 | G(A) | 0.0209 | 0.0021 | 0.3514 | 7.14×10^-23^ | 0.3052 | 0.00008273 | -0.0104 | 0.0200 | 0.2627 | 0.6044 |
| rs115288876 | 1 | A(G) | 0.0788 | 0.0050 | 0.0433 | 2.36×10^-56^ | 0.0368 | 0.00003569 | 0.0080 | 0.0449 | 0.0407 | 0.8590 |
| rs11542462 | 16 | A(G) | -0.0248 | 0.0030 | 0.1335 | 9.72×10^-17^ | 0.1511 | 0.00003563 | -0.0079 | 0.0303 | 0.0933 | 0.7936 |
| rs11600054 | 11 | A(G) | 0.0682 | 0.0101 | 0.0101 | 1.84×10^-11^ | 0.0109 | 0.00000196 | 0.0653 | 0.0633 | 0.0203 | 0.3025 |
| rs11726886 | 4 | A(C) | -0.0537 | 0.0023 | 0.2908 | 3.08×10^-125^ | 0.3333 | 0.00050672 | 0.0150 | 0.0189 | 0.3169 | 0.4297 |
| rs117300835 | 11 | A(G) | -0.3350 | 0.0089 | 0.0133 | 1.00×10^-200^ | 0.0202 | 0.00011386 | -0.1633 | 0.0809 | 0.0126 | 0.0436 |
| rs11791258 | 9 | A(G) | 0.0141 | 0.0026 | 0.1911 | 4.85×10^-8^ | 0.1364 | 0.00001412 | 0.0257 | 0.0265 | 0.1267 | 0.3316 |
| rs11867297 | 17 | T(C) | 0.0135 | 0.0021 | 0.3853 | 1.01×10^-10^ | 0.3788 | 0.00003959 | -0.0156 | 0.0185 | 0.3424 | 0.3993 |
| rs12056768 | 8 | G(T) | -0.0232 | 0.0021 | 0.5840 | 2.65×10^-29^ | 0.4245 | 0.00012417 | -0.0152 | 0.0176 | 0.5270 | 0.3884 |
| rs12283049 | 11 | G(A) | -0.0565 | 0.0024 | 0.2344 | 9.62×10^-122^ | 0.2495 | 0.00041489 | -0.0192 | 0.0223 | 0.1951 | 0.3894 |
| rs12324720 | 15 | A(G) | -0.0149 | 0.0027 | 0.1746 | 2.45×10^-8^ | 0.1948 | 0.00001963 | 0.0099 | 0.0210 | 0.2256 | 0.6375 |
| rs12462826 | 19 | A(G) | -0.0132 | 0.0021 | 0.3696 | 4.18×10^-10^ | 0.3549 | 0.00003596 | 0.0077 | 0.0186 | 0.3296 | 0.6807 |
| rs12501515 | 4 | A(G) | -0.0790 | 0.0021 | 0.5897 | 1.00×10^-200^ | 0.4374 | 0.00144187 | -0.0185 | 0.0181 | 0.6212 | 0.3056 |
| rs1260326 | 2 | C(T) | 0.0197 | 0.0021 | 0.6039 | 1.96×10^-21^ | 0.4105 | 0.00008802 | -0.0088 | 0.0184 | 0.6503 | 0.6329 |
| rs12775091 | 10 | T(C) | 0.0156 | 0.0025 | 0.2134 | 3.33×10^-10^ | 0.2416 | 0.00002911 | -0.0288 | 0.0193 | 0.2942 | 0.1358 |
| rs13108245 | 4 | G(A) | -0.0122 | 0.0021 | 0.3866 | 4.63×10^-9^ | 0.3887 | 0.00003284 | 0.0110 | 0.0183 | 0.3593 | 0.5467 |
| rs1321247 | 6 | T(A) | -0.0222 | 0.0034 | 0.1016 | 4.36×10^-11^ | 0.0859 | 0.00001373 | -0.0224 | 0.0278 | 0.1138 | 0.4212 |
| rs138335 | 22 | G(C) | -0.0138 | 0.0022 | 0.6586 | 1.56×10^-10^ | 0.3081 | 0.00003514 | -0.0294 | 0.0181 | 0.6328 | 0.1056 |
| rs1384687 | 8 | A(G) | -0.0169 | 0.0030 | 0.1323 | 1.82×10^-8^ | 0.1364 | 0.00001502 | 0.0090 | 0.0199 | 0.2647 | 0.6520 |
| rs142004400 | 14 | C(A) | -0.0310 | 0.0056 | 0.0342 | 3.01×10^-8^ | 0.0455 | 0.00000537 | -0.0648 | 0.0624 | 0.0206 | 0.2986 |
| rs142158911 | 19 | A(G) | 0.0263 | 0.0032 | 0.1118 | 4.43×10^-16^ | 0.096 | 0.00002306 | 0.0278 | 0.0288 | 0.1030 | 0.3333 |
| rs1532085 | 15 | G(A) | 0.0253 | 0.0021 | 0.6167 | 8.60×10^-34^ | 0.4091 | 0.00014284 | 0.0025 | 0.0177 | 0.5755 | 0.8858 |
| rs1627043 | 11 | C(G) | -0.0486 | 0.0057 | 0.0332 | 8.49×10^-18^ | 0.0303 | 0.00000873 | -0.0535 | 0.0463 | 0.0373 | 0.2480 |
| rs1684600 | 16 | T(C) | -0.0125 | 0.0022 | 0.2987 | 1.59×10^-8^ | 0.3586 | 0.00002956 | -0.0551 | 0.0203 | 0.2507 | 0.0066 |
| rs17207784 | 6 | C(T) | -0.0135 | 0.0022 | 0.3242 | 5.14×10^-10^ | 0.3384 | 0.00003480 | 0.0035 | 0.0189 | 0.3152 | 0.8516 |
| rs17473257 | 11 | A(G) | -0.0611 | 0.0078 | 0.0172 | 4.59×10^-15^ | 0.0253 | 0.00000610 | 0.0998 | 0.0693 | 0.0164 | 0.1499 |
| rs1800588 | 15 | T(C) | -0.0305 | 0.0025 | 0.2150 | 4.73×10^-35^ | 0.2020 | 0.00009899 | 0.0129 | 0.0203 | 0.2501 | 0.5257 |
| rs1858889 | 7 | C(A) | 0.0135 | 0.0020 | 0.5025 | 3.49×10^-11^ | 0.4747 | 0.00004404 | -0.0036 | 0.0176 | 0.4582 | 0.8370 |
| rs1871395 | 12 | G(A) | -0.0204 | 0.0028 | 0.1527 | 5.72×10^-13^ | 0.1465 | 0.00002614 | 0.0542 | 0.0197 | 0.2791 | 0.0058 |
| rs2037511 | 18 | A(G) | 0.0177 | 0.0027 | 0.1659 | 9.41×10^-11^ | 0.1616 | 0.00002287 | 0.0459 | 0.0239 | 0.1603 | 0.0555 |
| rs2074735 | 22 | C(G) | 0.0293 | 0.0041 | 0.0648 | 1.22×10^-12^ | 0.0505 | 0.00000974 | -0.0106 | 0.0253 | 0.1429 | 0.6738 |
| rs2229742 | 21 | C(G) | -0.0250 | 0.0033 | 0.1047 | 4.75×10^-14^ | 0.1111 | 0.00002259 | 0.0637 | 0.0278 | 0.1110 | 0.0221 |
| rs2297991 | 10 | C(T) | 0.0128 | 0.0023 | 0.7185 | 1.57×10^-8^ | 0.2828 | 0.00002610 | -0.0260 | 0.0185 | 0.6613 | 0.1612 |
| rs2494429 | 1 | G(A) | -0.0148 | 0.0027 | 0.8230 | 2.80×10^-8^ | 0.1616 | 0.00001682 | -0.0364 | 0.0232 | 0.8250 | 0.1171 |
| rs2511279 | 11 | G(C) | 0.0982 | 0.0052 | 0.9604 | 2.98×10^-79^ | 0.0455 | 0.00006210 | 0.0199 | 0.0347 | 0.9294 | 0.5665 |
| rs2595644 | 7 | T(G) | -0.0123 | 0.0021 | 0.3849 | 4.97×10^-9^ | 0.4242 | 0.00003362 | -0.0101 | 0.0185 | 0.3448 | 0.5851 |
| rs2710651 | 2 | A(G) | -0.0116 | 0.0020 | 0.5261 | 1.23×10^-8^ | 0.4798 | 0.00003259 | -0.0294 | 0.0177 | 0.4556 | 0.0961 |
| rs2756119 | 14 | A(G) | 0.0121 | 0.0021 | 0.3814 | 8.71×10^-9^ | 0.3333 | 0.00002961 | 0.0067 | 0.0181 | 0.3825 | 0.7099 |
| rs2807834 | 1 | G(T) | -0.0151 | 0.0022 | 0.6851 | 5.66×10^-12^ | 0.3131 | 0.00004107 | -0.0072 | 0.0194 | 0.7124 | 0.7089 |
| rs28435470 | 12 | A(G) | -0.0119 | 0.0021 | 0.6632 | 3.29×10^-8^ | 0.3638 | 0.00002844 | -0.0091 | 0.0179 | 0.5814 | 0.6101 |
| rs2847500 | 11 | A(G) | -0.0225 | 0.0031 | 0.1232 | 2.77×10^-13^ | 0.1263 | 0.00002370 | -0.0080 | 0.0249 | 0.1484 | 0.7483 |
| rs290400 | 20 | A(G) | -0.0131 | 0.0022 | 0.6655 | 1.41×10^-9^ | 0.3535 | 0.00003371 | 0.0145 | 0.0180 | 0.6130 | 0.4200 |
| rs3114045 | 4 | C(T) | -0.0222 | 0.0030 | 0.8661 | 1.00×10^-13^ | 0.1465 | 0.00002786 | -0.0020 | 0.0263 | 0.8707 | 0.9388 |
| rs325393 | 15 | T(G) | -0.0136 | 0.0023 | 0.2782 | 2.03×10^-9^ | 0.3030 | 0.00003055 | -0.0413 | 0.0190 | 0.3165 | 0.0296 |
| rs34186890 | 3 | G(A) | -0.0157 | 0.0023 | 0.2596 | 1.33×10^-11^ | 0.2475 | 0.00003431 | 0.0206 | 0.0203 | 0.2461 | 0.3121 |
| rs34726834 | 8 | T(C) | 0.0140 | 0.0023 | 0.2522 | 2.42×10^-9^ | 0.2576 | 0.00002740 | -0.0256 | 0.0192 | 0.2956 | 0.1834 |
| rs35270497 | 2 | T(C) | 0.0157 | 0.0027 | 0.1762 | 5.08×10^-9^ | 0.1768 | 0.00002001 | -0.0140 | 0.0215 | 0.2114 | 0.5158 |
| rs35823191 | 1 | C(T) | -0.0233 | 0.0021 | 0.3420 | 1.65×10^-27^ | 0.3182 | 0.00010311 | 0.0427 | 0.0182 | 0.3671 | 0.0192 |
| rs3732220 | 2 | A(G) | -0.0478 | 0.0036 | 0.0853 | 1.31×10^-39^ | 0.0909 | 0.00005768 | 0.0434 | 0.0332 | 0.0749 | 0.1909 |
| rs3829251 | 11 | A(G) | -0.1145 | 0.0030 | 0.1333 | 1.00×10^-200^ | 0.1919 | 0.00092028 | -0.0090 | 0.0198 | 0.2680 | 0.6491 |
| rs4348160 | 4 | G(T) | -0.0258 | 0.0022 | 0.3270 | 6.62×10^-33^ | 0.3737 | 0.00013448 | -0.0020 | 0.0176 | 0.4650 | 0.9099 |
| rs4364259 | 4 | A(G) | 0.0172 | 0.0026 | 0.1987 | 1.86×10^-11^ | 0.1566 | 0.00002398 | 0.0226 | 0.0214 | 0.2193 | 0.2908 |
| rs4420638 | 19 | G(A) | -0.0193 | 0.0027 | 0.1768 | 3.95×10^-13^ | 0.2172 | 0.00003604 | -0.0230 | 0.0199 | 0.2720 | 0.2464 |
| rs4580037 | 13 | C(A) | -0.0136 | 0.0023 | 0.2856 | 1.68×10^-9^ | 0.3081 | 0.00003115 | 0.0225 | 0.0198 | 0.2691 | 0.2544 |
| rs512083 | 1 | C(T) | 0.0122 | 0.0020 | 0.4625 | 2.23×10^-9^ | 0.4646 | 0.00003581 | 0.0061 | 0.0175 | 0.4872 | 0.7286 |
| rs5770794 | 22 | T(C) | -0.0133 | 0.0022 | 0.3143 | 1.74×10^-9^ | 0.2980 | 0.00003052 | 0.0013 | 0.0182 | 0.3687 | 0.9427 |
| rs6129648 | 20 | G(A) | 0.0141 | 0.0021 | 0.3798 | 2.44×10^-11^ | 0.3535 | 0.00004100 | 0.0131 | 0.0185 | 0.3351 | 0.4794 |
| rs61698755 | 17 | C(T) | -0.0115 | 0.0021 | 0.5600 | 2.25×10^-8^ | 0.4394 | 0.00003099 | -0.0066 | 0.0177 | 0.5500 | 0.7103 |
| rs61747728 | 1 | T(C) | 0.0303 | 0.0053 | 0.0386 | 8.83×10^-9^ | 0.0303 | 0.00000391 | 0.0235 | 0.0365 | 0.0631 | 0.5196 |
| rs61813875 | 1 | G(C) | 0.0821 | 0.0066 | 0.0248 | 1.16×10^-35^ | 0.0202 | 0.00001238 | 0.0593 | 0.0968 | 0.0087 | 0.5401 |
| rs61887421 | 11 | C(T) | -0.0367 | 0.0060 | 0.0301 | 8.05×10^-10^ | 0.0152 | 0.00000227 | -0.0277 | 0.0511 | 0.0308 | 0.5877 |
| rs62129966 | 19 | A(C) | 0.0612 | 0.0028 | 0.1609 | 1.60×10^-108^ | 0.0231 | 0.00000000 | -0.0189 | 0.0244 | 0.1528 | 0.4378 |
| rs6438900 | 3 | G(C) | 0.0150 | 0.0023 | 0.2560 | 1.27×10^-10^ | 0.2879 | 0.00003412 | 0.0070 | 0.0191 | 0.3057 | 0.7126 |
| rs6672758 | 1 | T(C) | 0.0162 | 0.0026 | 0.8002 | 2.04×10^-10^ | 0.2121 | 0.00002719 | -0.0254 | 0.0200 | 0.7420 | 0.2033 |
| rs6834488 | 4 | T(C) | -0.0145 | 0.0021 | 0.4228 | 2.26×10^-12^ | 0.4141 | 0.00004809 | 0.0190 | 0.0187 | 0.3241 | 0.3108 |
| rs71599974 | 4 | G(A) | 0.0257 | 0.0029 | 0.1480 | 2.39×10^-19^ | 0.1465 | 0.00004070 | 0.0059 | 0.0256 | 0.1360 | 0.8168 |
| rs733454 | 11 | T(C) | 0.0189 | 0.0034 | 0.0992 | 2.93×10^-8^ | 0.1061 | 0.00001174 | 0.0291 | 0.0383 | 0.0569 | 0.4475 |
| rs73413596 | 12 | C(T) | 0.0223 | 0.0039 | 0.0740 | 9.15×10^-9^ | 0.0808 | 0.00000987 | 0.0200 | 0.0374 | 0.0577 | 0.5928 |
| rs742493 | 6 | C(T) | 0.0184 | 0.0032 | 0.1129 | 1.04×10^-8^ | 0.1768 | 0.00001919 | 0.0103 | 0.0296 | 0.0975 | 0.7267 |
| rs7528419 | 1 | G(A) | 0.0215 | 0.0024 | 0.2244 | 8.17×10^-19^ | 0.2828 | 0.00006405 | -0.0159 | 0.0214 | 0.2159 | 0.4587 |
| rs7569755 | 2 | A(G) | 0.0136 | 0.0023 | 0.2886 | 1.49×10^-9^ | 0.3182 | 0.00003191 | 0.0121 | 0.0213 | 0.2160 | 0.5709 |
| rs7580771 | 2 | T(G) | -0.0166 | 0.0027 | 0.1760 | 5.15×10^-10^ | 0.1616 | 0.00002106 | -0.0100 | 0.0210 | 0.2232 | 0.6333 |
| rs7712001 | 5 | G(T) | 0.0119 | 0.0021 | 0.4402 | 7.05×10^-9^ | 0.4343 | 0.00003314 | -0.0461 | 0.0181 | 0.3782 | 0.0111 |
| rs77924615 | 16 | A(G) | -0.0152 | 0.0026 | 0.1944 | 3.94×10^-9^ | 0.1970 | 0.00002206 | -0.0088 | 0.0212 | 0.2231 | 0.6781 |
| rs77960347 | 18 | G(A) | -0.0526 | 0.0091 | 0.0127 | 6.53×10^-9^ | 0.0253 | 0.00000334 | -0.0166 | 0.1014 | 0.0074 | 0.8702 |
| rs78649910 | 4 | A(T) | -0.0191 | 0.0033 | 0.1057 | 8.33×10^-9^ | 0.0859 | 0.00001049 | -0.0214 | 0.0270 | 0.1212 | 0.4282 |
| rs7955128 | 12 | T(A) | 0.0131 | 0.0020 | 0.5205 | 1.48×10^-10^ | 0.4040 | 0.00003978 | 0.0060 | 0.0178 | 0.5621 | 0.9735 |
| rs8018720 | 14 | C(G) | -0.0345 | 0.0027 | 0.8235 | 1.94×10^-38^ | 0.1414 | 0.00008213 | -0.0077 | 0.0246 | 0.8516 | 0.7548 |
| rs8107974 | 19 | T(A) | 0.0356 | 0.0038 | 0.0763 | 1.36×10^-20^ | 0.0909 | 0.00002878 | -0.0336 | 0.0356 | 0.0646 | 0.3456 |
| rs8121940 | 20 | G(C) | -0.0436 | 0.0025 | 0.1976 | 1.77×10-65 | 0.2172 | 0.00019985 | -0.0155 | 0.0209 | 0.2324 | 0.4580 |
| rs9375037 | 6 | C(A) | 0.0117 | 0.0021 | 0.4432 | 1.21×10^-8^ | 0.4343 | 0.00003211 | 0.0153 | 0.0176 | 0.4622 | 0.3850 |
| rs9409266 | 9 | A(G) | -0.0168 | 0.0029 | 0.8622 | 1.24×10^-8^ | 0.1616 | 0.00001768 | 0.0263 | 0.0233 | 0.8289 | 0.2587 |
| rs964184 | 11 | C(G) | 0.0407 | 0.0030 | 0.8672 | 3.50×10^-42^ | 0.1364 | 0.00008781 | -0.0475 | 0.0248 | 0.8541 | 0.0549 |
| rs9847248 | 3 | A(G) | -0.0123 | 0.0022 | 0.7130 | 4.19×10^-8^ | 0.2879 | 0.00002480 | 0.0581 | 0.0185 | 0.6545 | 0.0017 |
| rs986649 | 5 | G(A) | 0.0129 | 0.0022 | 0.3216 | 3.51×10^-9^ | 0.3333 | 0.00003119 | 0.0016 | 0.0181 | 0.3786 | 0.9284 |
| rs9946771 | 18 | T(C) | -0.0234 | 0.0041 | 0.0663 | 9.47×10^-9^ | 0.0909 | 0.00001096 | 0.0397 | 0.0325 | 0.0783 | 0.2221 |

Abbreviations: Chr = chromosome; EAF = effect allele frequency; MAF = minor allele frequency; SE = standard error.
