# Supplementary material for: Causal effects of endometriosis on serum 25-hydroxyvitamin D: Evidence from Mendelian randomization study
Source: Medicine (Baltimore). 2026 May 8;105(19):e48562. doi: 10.1097/MD.0000000000048562 (PMC13166774; doi:10.1097/MD.0000000000048562)
Supplement: Supplementary file 2 [file medi-105-e48562-s002.docx]

**TABLE S2. Single-Nucleotide Polymorphisms (SNPs) Linked to Endometriosis and Their Association with the Levels of 25-Hydroxyvitamin D**

| **Target SNP** | **Chr** | **Effect Allele (Alternative)** | **Association with endometriosis** | | | | **MAF** | **R^2^** | **Association with 25-hydroxyvitamin D levels** | | | |
| --- | --- | --- | --- | --- | --- | --- | --- | --- | --- | --- | --- | --- |
|  |  |  | **β** | **SE** | **EAF** | ***p*** |  |  | **β** | **SE** | **EAF** | ***p*** |
| rs10757277 | 9 | G(A) | -0.1013 | 0.0178 | 0.4203 | 1.25×10^-8^ | 0.4742 | 0.00003731 | -0.0008 | 0.0020 | 0.4827 | 0.7095 |
| rs11031005 | 11 | C(T) | -0.2379 | 0.0239 | 0.1676 | 2.63×10^-23^ | 0.0960 | 0.00001095 | -0.0017 | 0.0029 | 0.1426 | 0.5625 |
| rs140993356 | 6 | G(A) | 0.3247 | 0.0379 | 0.0588 | 1.01×10^-17^ | 0.0202 | 0.00000292 | 0.0080 | 0.0058 | 0.0321 | 0.1642 |
| rs17694933 | 9 | A(G) | 0.1331 | 0.0177 | 0.4243 | 4.71×10^-14^ | 0.4141 | 0.00004143 | -0.0010 | 0.0021 | 0.4326 | 0.6265 |
| rs28517654 | 4 | C(T) | -0.1356 | 0.0205 | 0.2432 | 3.86×10^-11^ | 0.2879 | 0.00003116 | -0.0021 | 0.0023 | 0.2794 | 0.3475 |
| rs6546324 | 2 | C(A) | -0.1250 | 0.0190 | 0.6921 | 4.31×10^-11^ | 0.3256 | 0.00006197 | -0.0013 | 0.0022 | 0.6937 | 0.5456 |
| rs7455028 | 6 | T(C) | 0.1247 | 0.0184 | 0.3428 | 1.13×10^-11^ | 0.0275 | 0.00004853 | -0.0009 | 0.0023 | 0.2734 | 0.6772 |

Abbreviations: Chr = chromosome; EAF = effect allele frequency; MAF = minor allele frequency; SE = standard error.
